# Reduction of leakage from insertion site during continuous femoral nerve block with catheter-through-needle versus catheter-over-needle technique for postoperative analgesia after total knee arthroplasty: a randomized controlled trial

**DOI:** 10.1186/s12871-021-01554-9

**Published:** 2022-01-05

**Authors:** Yoshiyasu Hattammaru, Yasushi Mio, Tomasz Hascilowicz, Isao Utsumi, Yuichi Murakami, Sachiko Omi

**Affiliations:** Department of Anesthesiology, Jikei University School of Medicine, 3-25-8 Nishi-shinbashi, Minato-ku, Tokyo, 105-8461 Japan

**Keywords:** continuous femoral nerve block, catheter-over-needle, leakage from catheter insertion site, total knee arthroplasty

## Abstract

**Background:**

Continuous femoral nerve block (CFNB) is a common procedure used for postoperative analgesia in total knee arthroplasty. Continuous nerve block using a conventional needle (catheter-through-needle/CTN) is complicated by leakage of the anesthetic from the catheter insertion site. A different type of needle (catheter-over-needle/ CON) is now available, which is believed to reduce leakage as the diameter of the catheter is larger than that of the needle. The purpose of this study was to compare the incidence of leakage from the catheter insertion site during CFNB while using CTN and CON for postoperative analgesia after total knee arthroplasty (TKA).

**Methods:**

This prospective, randomized, single-blinded controlled study included 60 patients who were scheduled for TKA at our facility between May 2016 and November 2017. Patients were randomly allocated to the CTN or CON groups. All patients in both groups received CFNB and sciatic nerve block for postoperative analgesia. The administration of 0.16% levobupivacaine mixed with 6 mg of indigo carmine (a dye added to easily identify leakage) was started at 6 ml/h at the end of surgery. The primary outcome was the incidence of leakage from the catheter insertion site. We further investigated the degree of leakage, the incidence of catheter migration, pain scores using the numerical rating scale at 48 h postoperatively, and the number of days until the operated knee could be flexed 120 degrees postoperatively in both groups.

**Results:**

The CON group had a significantly lower incidence and degree of leakage from the catheter insertion site. There were no significant differences in other measurement outcomes.

**Conclusions:**

Use of CON reduces the incidence of leakage from the catheter insertion site during CFNB in the use of postoperative analgesia for total knee arthroplasty. Future research is needed to determine additional benefits of using CON related to decreased leakage.

**Trial registration:**

The study was registered in the University Hospital Medical Information Network (UMIN) Clinical Trials Registry (UMIN000021537), prospectively registered on 18 March 2016.

## Background

In recent years, ultrasound-guided nerve blocks have partly replaced epidural anesthesia, and catheter placement for continuous peripheral nerve block (CPNB) has been used in invasive surgeries. CPNB can provide optimal analgesia via titration of the optimal amount and concentration of local anesthetic through the catheter. CPNB improves postoperative analgesia and decreases supplemental analgesic requirements using interscalene, paravertebral, adductor canal, femoral, and sciatic catheters [1]. Particularly, continuous femoral nerve block (CFNB) for total knee arthroplasty (TKA) reduced morphine consumption, pain score, and hospital stay and improved knee range of motion [2]. However, CPNB performed with a conventional catheter-through-needle (CTN) is associated with leakage of anesthetic from the catheter insertion site, which may lead to catheter malposition and attenuation of analgesic effects [3–5]. Catheter-over-needle (CON), a needle for CPNB has been made available. The advantage of CON is considered to be the lower level of leakage from the catheter insertion site compared with CTN because the diameter of the catheter is larger than that of the needle. Although several randomized clinical trials have been conducted on CTN versus CON, there have been very few trials comparing the efficacy and safety of CTN and CON [6–9].

This study investigated whether there was a difference in the incidence of leakage from the catheter insertion site of the CFNB between CTN and CON when used for TKA.

## Methods

### Data Collection

Patients aged 20–80 years who were scheduled for monolateral TKA from May 11, 2016 to November 8, 2017 were included in this trial. The exclusion criteria included re-operation of TKA, local anesthetic allergy, infection at the insertion site, American Society of Anesthesia physical status > III, daily steroid use (prednisolone > 20 mg/day), and inappropriate entry into the study based on the judgment of the investigator. Sixty consecutive TKA patients, from whom informed consent was obtained and who fulfilled the inclusion criteria were enrolled.

Sixty patients were randomized into two groups using Microsoft Excel (Redmond, WA, USA): the CTN and CON groups. Contiplex (B. Braun, Meisungen, Germany) and Contiplex C (B. Braun, Meisungen, Germany) were used in this study in the CTN and CON groups, respectively. The randomized table was kept by an independent researcher (Y Murakami) who was not involved in anesthesia procedures or outcome assessments.

### Anesthetic Procedure

All nerve blocks were performed by YH and SO, who have substantial experience in performing ultrasound-guided nerve blocks. The Edge2 ultrasound system linear probe (HFL50x, 6–15 MHz) (Sonosite, Bothell, USA) and Stimplex HNS12 nerve stimulator system (B. Braun, Meisungen, Germany) were used. In each case, postoperative follow-up and evaluation were performed by the same anesthesiologist who conducted the nerve blocks.

Noninvasive blood pressure, electrocardiogram, heart rate, and pulse oxygen saturation were monitored before and during the femoral nerve block procedure. The femoral nerve was identified in the inguinal region with a transverse cross-sectional view under ultrasound guidance. The needle tip was advanced to the dorsal side of the femoral nerve near the femoral artery, and the tip position was confirmed after administration of saline. The catheter was placed 5 cm from the needle tip. After placement of the catheter, a local anesthetic (0.25% ropivacaine, 10 ml) was administered through the catheter. A sciatic nerve block was then performed through the popliteal approach. General or spinal anesthesia were selected as intraoperative anesthesia. At the end of surgery, 10 ml of 0.125% levobupivacaine was administered through the catheter, and 300 ml of 0.16% levobupivacaine mixed with 6 mg (1.5 ml) of indigo carmine was started at 6 ml/h using a COOPDECH Balloonjector infusion pump (Daiken iki, Osaka, Japan). Indigo carmine was used as a dye to easily identify the leakage of anesthetics. For the use of indigo carmine, we referred to the literature on its use in surgery [10]. The catheter insertion site was covered with a 7 cm diameter white filter paper to observe local anesthetic leakage. Changes in dressing at the insertion site were prohibited until the catheter was removed. Forty-eight hours after completion of the surgery, the catheter was removed and the degree of staining with indigo carmine was assessed. Postoperative analgesia other than CFNB included celecoxib 200 mg daily at regular doses and acetaminophen up to 2400 mg daily as needed.

### Measurement outcome

**Fig. 1:**
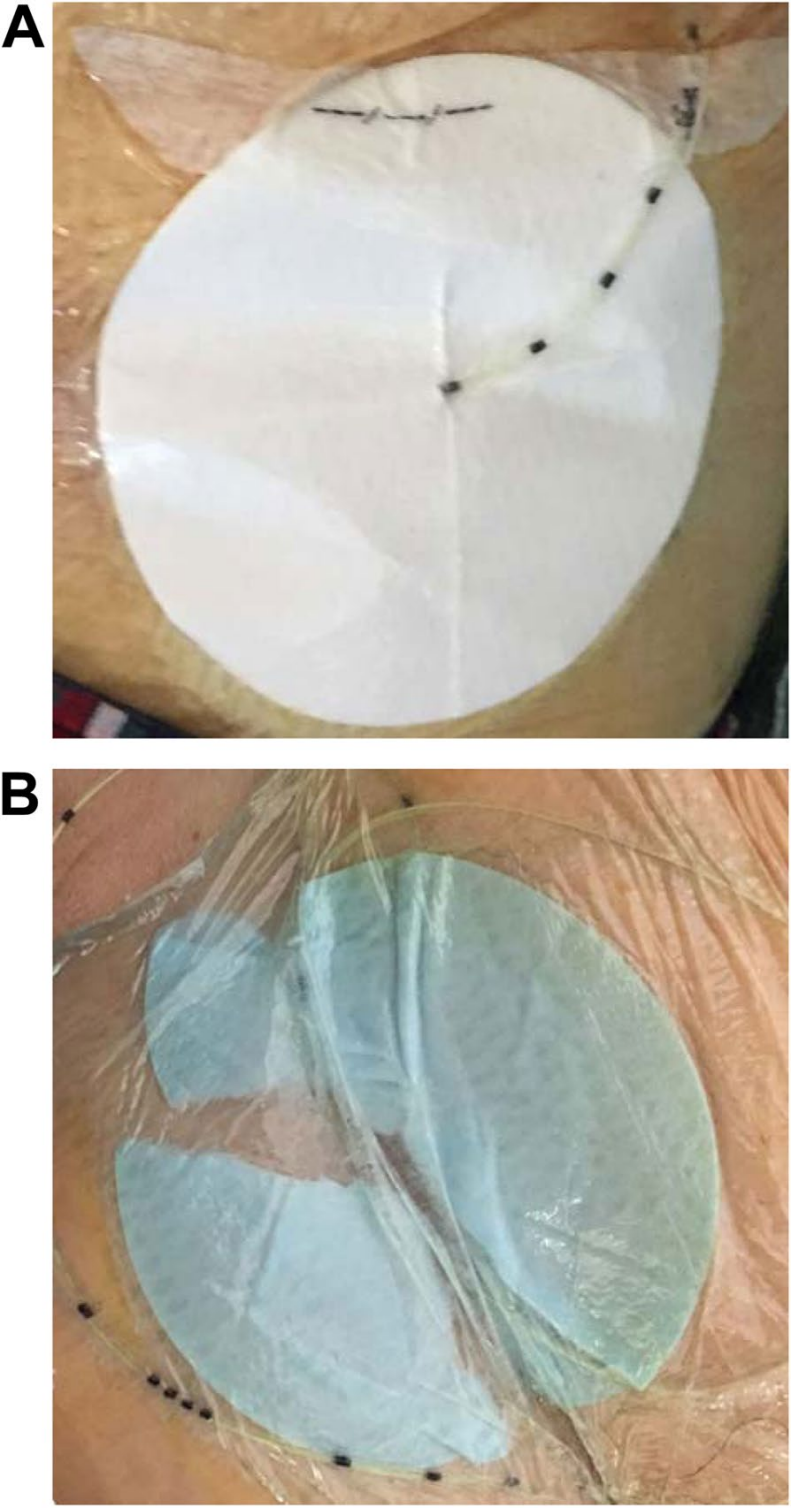
Catheter insertion site 48 h after the start of continuous femoral nerve block. A: No leakage. White filter paper was not dyed at all. B: The filter paper was completely dyed blue and the soaked paper was torn. The leakage could be identified easily by adding indigo carmine to local anesthetics

The primary outcome was the incidence of anesthetic leakage. Leakage was clearly identified with indigo carmine. We defined leakage as that occurring when any amount of blue dye was observed on the filter paper. No leakage was defined by the absence of blue dye on the filter paper (Fig. 1A). Secondary outcomes were the incidence at which the 7 cm diameter filter paper was dyed completely in the cases of leakage, the incidence of catheter malposition, pain scores using the Numerical Rating Scale (NRS) at 48 h postoperatively, and the number of days until the operated knee could be flexed 120° postoperatively. Catheter malposition was defined as migration of > 1 cm or more. Additionally, we investigated the presence of complications related to nerve blocks, such as nerve injury, bleeding, infection, and local anesthetic systemic toxicity.

### Statistical Analysis

The sample size was calculated based on a previous study [7] using the Power and Sample Size Program [11], with an alpha error of 0.05, a detection rate of 0.8, a leak rate of 80% for the CTN group, and a leak rate of 30% for the CON group. All statistical analyses were conducted using the IBM SPSS Statistics software version 27 (IBM Corp., Armonk, NY, USA). Fisher’s exact test and Wilcoxon rank sum test were conducted to compare each measurement outcome between the CTN and CON groups. Statistical significance was set at p < 0.05.

## Results

There were no differences in age, sex, body mass index, and anesthetic method (Table 1). Leakages were not present before the start of continuous administration of the local anesthetic in all cases. One patient in the CTN group was excluded from the statistical analysis of measurement outcomes because the catheter was disconnected from the infusion pump before completion of 48 h of continuous local anesthetic administration. Leakage was significantly larger in the CTN group. In 6 cases in the CON group and 25 cases in the CTN group, a 7 cm diameter filter paper was dyed completely (Fig. 1B). Five out of 27 leaks in the CTN group and 2 out of 10 leaks in the CON group resulted in a shortened catheter insertion length. There was no significant difference in the number of days until the knee joint could be flexed to 120° after surgery or in the NRS 48 h after surgery (Table 2). None of the nerve block-related complications were observed in any of the patients.

**Table 1:** Clinical characteristics of patients

Characteristic	CTN group	CON group	*P* value
Number of patients	30	30	
Age, yr	71 ± 6	71 ± 9	0.60
Male / Female, n	5 / 25	7 / 23	0.75
BMI	27.1 ± 4.4	26.5 ± 4.1	0.66
GA / SA, n	2 / 28	1 / 29	0.55

Values are mean ± SD or number of patients.

*Abbreviations*: *BMI* body mass index, *CTN* catheter-through-needle, *CON* catheter-over-needle, *GA* general anesthesia, *SA* spinal anesthesia

**Table 2:** Measurement outcomes

	CTN group	CON group	*P* value
Number of patients	29	30	
Incidence of leakage, n	27	10	< 0.001
Incidence at which the filter paper dyed blue completely, n	25	6	< 0.001
Incidence of catheter malposition, n	5	2	0.20
Pain score at rest after 48 h, median [range]	1 [0–5]	2 [0–6]	0.38
Postoperatively days until the knee could be flexed 120 degrees, mean ± SD	5.9 ± 4.0	7.3 ± 4.0	0.13

*Abbreviations*: *CTN* catheter-through-needle, *CON* catheter-over-needle

## Discussion

This randomized study compared the differences between CTN and CON in regard to the incidence of leakage from the insertion site following CFNB for postoperative analgesia in TKA. The incidence of leakage from the insertion site and the rate at which the filter paper was completely dyed blue were lower in the CON group than in the CTN group. Other secondary endpoints did not differ between the two groups.

TKA is associated with moderate to severe postoperative pain. Inadequate analgesia can lead to complications such as deep vein thrombosis, pneumonia, and pulmonary embolism, resulting in delayed rehabilitation and prolonged hospital stay [12]. Early achievement of a 120-degree knee flexion position by passive rehabilitation after TKA improves performance of activity of daily living (ADL) [13]. Currently, the combination of femoral nerve block and sciatic nerve block, the method used in this study, is considered the best postoperative analgesic method for TKA [14]. There is no clear guideline on whether femoral nerve block should be administered as a single dose or as a continuous dose during TKA [15]. We believe that postoperative pain is the most severe at the start of rehabilitation, and we prefer to perform CFNB. One of the problems in performing CPNB is the leakage of local anesthetic from the catheter insertion site. The use of glue at the puncture site [8, 16] and tunneling of catheters [17] have been reported to be effective in preventing leakage; however, both of these methods require extra costs and invasive procedures.

In this study, the incidence of leakage was significantly lower in the CON group than in the CTN group. Yu [7] and Nogawa [9] reported that there were no leaks during catheter insertion using a CON needle. Our results showed 10 leaks out of 30 CON cases, and the incidence of leaks was higher when using CON needles than in the previous studies. In our study, leak detection was sensitive due to the addition of dye to the local anesthetic and placing a white filter paper on the puncture site. This may have made it easier to detect leakage compared to previous studies, in which leakage was assessed by visual observation alone. In addition, Nogawa et al. [9] performed a femoral nerve block with a single-use needle and then used a CON needle to place a catheter for CFNB. The use of two different needles may have influenced the leakage from the puncture site.

A 7 cm diameter filter paper was dyed completely in 60% (6 patients) of leakage cases in the CON group, and 93% (25 patients) of leakage cases in the CTN group. The amount of leakage was higher in cases where the filter paper was dyed completely than in cases with partially dyed filter paper. This indicates that the degree of leakage was greater in the CTN group than in the CON group. Previous reports have only detected leakage, whereas our evaluation of degree was qualitative, showing the differences in the degree of leakage between CTN and CON using dye.

There were no differences in the occurrence of catheter malposition, postoperative pain scores, and the number of days to achieve 120 degrees of knee joint flexion in the passive rehabilitation between the CTN and CON groups. Since the incidence and degree of anesthetic leakage from the insertion site were significantly higher in the CTN group, it is possible that the leakage does not affect catheter fixation, postoperative analgesic effects, and ADL performance after surgery. The cost of CON in our country is approximately 15 US dollars higher than that of CTN. Reducing leakage via CON may reduce the frequency at which patients’ clothes become wet and the dressing changes. This leads to a decrease in complaints from patients and nurses. CPNBs are placed as part of outpatient surgery [18]. The use of CON may be suitable for outpatients because of reduced leakage from the catheter insertion site. Further studies are needed to assess the advantages of using CON commensurate with cost. Advantages may include increase in inpatient and outpatient satisfaction as well as easier management of catheter insertion sites.

In contrast to previous reports [7, 9], in this study, opioids were not used for postoperative analgesia. The combination of a single preoperative sciatic nerve block followed by a CFNB and postoperative use of oral nonsteroidal anti-inflammatory drugs (NSAIDs) or acetaminophen could provide effective analgesia after surgery (pain score: median 2 and 1 for CTN and CON groups, respectively). This may indicate that the use of opioids can be avoided for postoperative analgesia in TKA. Since the use of opioids induces vomiting and can cause tolerance and withdrawal symptoms even after a short period of use, our results are also useful to consider the possibility of avoiding the use of postoperative opioids [19].

### Study limitations

Because the nerve block was performed by two practitioners, it is possible that the same technique was not used to place a CFNB catheter in all patients. In addition, we did not measure the time required for the procedure. The technical difficulties in inserting a catheter for CFNB may not be the same in the CTN and CON groups. There was no significant difference in BMI between the two groups; however, we did not compare the thickness of subcutaneous fat at the puncture site, which may have influenced the results. The use of filter paper at the puncture site, which is not commonly used, might have influenced leakage from the puncture site or catheter malposition. The degree of leakage was not assessed quantitatively, but was instead assessed qualitatively, because the 7 cm filter paper was completely dyed blue in most leakage cases.

## Conclusions

When CFNB was used for analgesia in TKA, the use of CON resulted in less frequent and a lower degree of anesthetic leakage from the insertion site than the use of CTN. Further studies are needed to examine the benefits of reducing leakage by using CON in patients and healthcare workers.

## Data Availability

The datasets used and analyzed during the present study are available from the first author on reasonable request.

## Abbreviations

ADL: Activities of Daily Living
CFNB: Continuous femoral nerve block
CON: Catheter-over-needle
CPNB: Continuous peripheral nerve block
CTN: Catheter-through-needle
NRS: Numerical Rating Scale
NSAID: Nonsteroidal anti-inflammatory drug
TKA: Total knee arthroplasty
